# Active remote monitoring of long-term conditions with mobile devices: a systematic review of cost-effectiveness analyses

**DOI:** 10.1038/s41746-025-01898-3

**Published:** 2025-10-24

**Authors:** Sean P. Gavan, Katherine Payne, William G. Dixon, Sabine N. van der Veer, Alexander C. T. Tam, Nick Bansback

**Affiliations:** 1 Manchester Centre for Health Economics, Division of Population Health, Health Services Research and Primary Care, School of Health Sciences, Faculty of Biology, Medicine and Health, The University of Manchester, Manchester, UK; 2 NIHR Manchester Biomedical Research Centre, Manchester Academic Health Science Centre, Manchester University NHS Foundation Trust, Manchester, UK; 3 Centre for Epidemiology Versus Arthritis, Division of Musculoskeletal and Dermatological Sciences, School of Biological Sciences, Faculty of Biology Medicine and Health, The University of Manchester, Manchester, UK; 4 Centre for Health Informatics, Division of Informatics, Imaging and Data Science, School of Health Sciences, Faculty of Biology Medicine and Health, The University of Manchester, Manchester, UK; 5 Centre for Advancing Health Outcomes, Providence Research Institute, St Paul’s Hospital, Vancouver, BC Canada; 6 School of Population and Public Health, University of British Columbia, Vancouver, BC Canada; 7 Honorary Professor, Division of Musculoskeletal and Dermatological Sciences, School of Biological Sciences, Faculty of Biology, Medicine and Health, The University of Manchester, Manchester, UK

**Keywords:** Health care economics, Signs and symptoms

## Abstract

This study aimed to identify and appraise published cost-effectiveness analyses of mobile device-based active remote monitoring technologies for long-term conditions. A systematic literature review (PROSPERO: CRD42023406364) identified studies from Medline and Embase (2008 until November 2024). Interventions required frequent patient-reported responses to questions about their condition on a mobile device (smartphone or tablet). Seven cost-effectiveness analyses were identified for six long-term conditions: rheumatoid arthritis; schizophrenia; older adults with complex conditions; cancer; multiple sclerosis; inflammatory bowel disease. Interventions facilitated early intervention to prevent condition worsening (*n* = 4); self-management (*n* = 2); and patient-initiated care (*n* = 1). Intervention costs were estimated by top-down costing (*n* = 2); bottom-up micro-costing (*n* = 3) and assumptions (*n* = 2). Mobile device-based active remote monitoring was cost-effective in six of the seven studies with a high degree of decision uncertainty. The results will help decision-makers, intervention developers and analysts to guide resource allocation, product development and study designs for future mobile device-based monitoring interventions, respectively.

## Introduction

Long-term conditions comprise those which last for at least 1 year and impact a person’s life (for example, arthritis, diabetes, epilepsy and heart disease)^1^. Incomplete information about health status or disease changes over time leads to challenges in managing people with long-term conditions. For example, imperfect recall of symptoms at follow-up appointments spaced at regular intervals (every 6-months or 12-months) may hamper the quality of clinical decisions, the ability to facilitate shared decision-making and opportunities may be missed to prevent worsening outcomes^2^. Similarly, if people are experiencing low and stable symptoms, then less resource-intensive management strategies may be possible for these individuals within a patient-initiated follow-up model of service delivery^3^. Rapid access to tailored self-management advice is also often needed to help people with long-term conditions immediately from the time at which symptoms occur^4^. Digital technologies that use electronic patient-generated health data are now emerging as one potential solution to overcome these challenges^5,6^.

Digital remote monitoring technologies that facilitate regular measurements of important outcomes or associated health data over time are experiencing a rapid growth internationally^7^. Passive remote monitoring technologies collect health data without direct user input through wearable devices or sensor technologies^8^. However, collecting data by passive remote monitoring alone is constrained by the hardware available and the clinical interpretability of the reported metrics, which narrows the scope of long-term conditions amenable to these interventions^9^. For example, continuous glucose monitoring is clinically useful in diabetes, while a step count is much harder to interpret in arthritis because it is influenced by more than just disease severity. Active remote monitoring technologies, by contrast, comprise direct user input to record measurements of symptoms and other patient-reported outcomes at regular time intervals (for example, daily, weekly, or monthly measurements)^10^. These technologies can be designed to collect self-reported data items that are most relevant to inform management decisions as they begin to improve or worsen over time (such as self-reported pain, functional ability, fatigue, mental wellbeing, or broader quality of life dimensions)^10^. The high prevalence of mobile device (smartphone and tablet) ownership provides significant scope to deploy app-based active remote monitoring technologies at scale at a relatively low marginal cost per user without the need for additional external devices^11^.

Care providers will often face an upfront cost to roll out any digital technology for their patient populations^12^. These upfront costs will vary by context, including the need for digital infrastructure (bespoke digital platforms or integration with an existing platform), licence fees to access software or reporting systems (per-user or block contracts), and staffing requirements (user support, ongoing maintenance and technical updates)^13^. Greater digital integration may also change downstream costs by amending established care pathways or practices^14^. As a result of these costs, healthcare payers and decision-makers typically require evidence to demonstrate the value of digital health technologies before their wider adoption^14,15^. Robust evidence for the potential cost and health benefits will be essential to support the wider adoption of mobile device-based active remote monitoring across populations with long-term conditions^16^.

Frameworks to assess the value of digital health technologies are now being used by healthcare decision-makers globally. Most notably, the National Institute for Health and Care Excellence in England have adopted an evidence standards framework for digital health technologies^17^, the Institute for Clinical and Economic Review and Peterson Health Technology Institute have an assessment framework for digital health technologies in the USA^18^, and the World Bank have published their framework for the economic evaluation of digital health interventions^19^. Cost-effectiveness analysis is a core method of economic evaluation to support healthcare resource allocation decisions^20^. A cost-effectiveness analysis performs an incremental comparison of a new health technology with a relevant comparator in terms of the difference in cost incurred and health outcomes achieved^20^. Cost-effectiveness analyses can be embedded alongside a randomised controlled trial (RCT) or may use a decision-analytic modelling framework to simulate cost and health outcomes per patient over a longer time horizon^21,22^. No study to date has undertaken a detailed appraisal of cost-effectiveness analyses designed specifically for mobile device-based active remote monitoring strategies that require regular user input to create electronic patient-generated health data over time.

Cost-effectiveness analyses of mobile device-based active remote monitoring strategies need to show how frequent and sustained user responses to self-reported questions about a long-term health condition influence measurable health outcomes and/or costs to the healthcare system. Possible channels through which these active remote monitoring interventions can demonstrate value include improving health outcomes directly (for example, by offering timely self-management advice which delivers an immediate health benefit); improving health outcomes indirectly (for example, by improving the effectiveness of subsequent clinical decisions due to the availability of longitudinal patient-reported data); or reducing healthcare resource use (for example, by substituting outpatient visits to monitor a condition with a remote digital alternative). To support healthcare decision-makers and mobile app developers, this study aimed to identify and appraise published cost-effectiveness analyses of mobile device-based active remote monitoring strategies for long-term conditions.

## Results

**Fig. 1:**
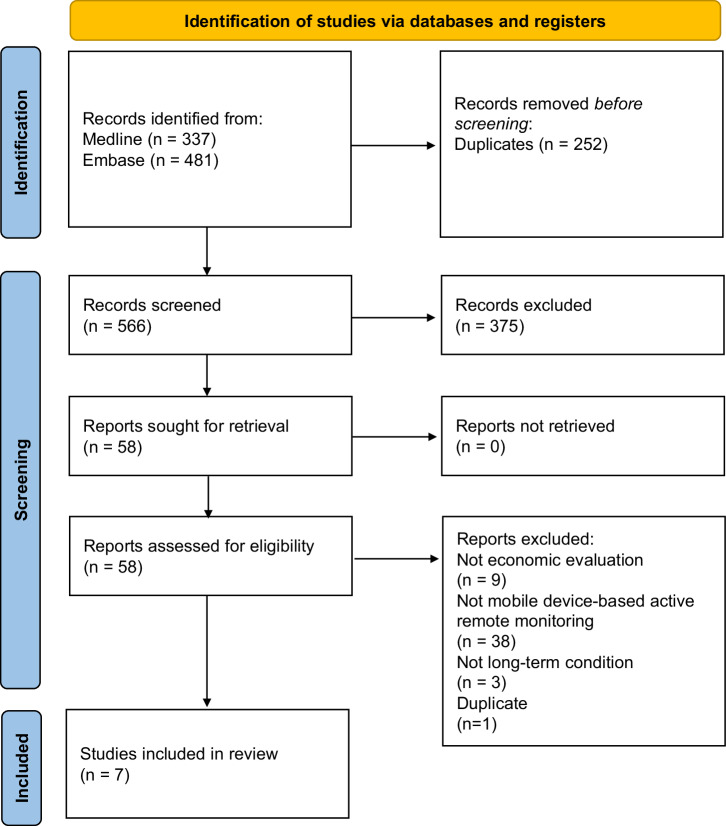
PRISMA flow diagram. The PRISMA flow diagram reports the abstract screening and full text review processes to achieve the final sample of seven cost-effectiveness analyses. The template PRISMA flow diagram is available from the original source publication with a Creative Commons Attribution (CC BY 4.0) license^23^.

Figure 1 reports a PRISMA flow diagram to illustrate the study inclusion process^23^. Cohen’s kappa was 0.73, which indicated substantial agreement between the two authors at the abstract screening stage. The database search identified 566 unique records. The final sample comprised seven published cost-effectiveness analyses of mobile device-based active remote monitoring strategies^24–30^.

### Study design characteristics

Table 1 summarises the overall design of each study to assess the cost-effectiveness of each intervention. All studies were published recently (year published: 2020, *n* = 1; 2021, *n* = 2; 2022, *n* = 3; 2023, *n* = 1). The target populations comprised six different long-term conditions: rheumatoid arthritis (*n* = 2)^24,28^, schizophrenia (*n* = 1)^27^; older individuals with complex chronic conditions (*n* = 1)^29^; cancer (*n* = 1)^30^; multiple sclerosis (*n* = 1)^25^; and inflammatory bowel disease (*n* = 1)^26^. These target populations were further categorised by having ongoing active disease (*n* = 1)^24^ or stable disease activity/recent recovery (*n* = 3)^27,28,30^. Three studies did not specify the degree of disease activity or severity within their target populations^25,26,29^.

**Table 1:** Summary characteristics of the cost-effectiveness analyses

Author (Year)	Country	Target population	Type of analysis	Study design	Sample size
Bernard et al.^24^	France	Rheumatoid arthritis with moderate-to-high disease activity	CUA	Single centre RCT	89
Cloosterman et al.^25^	The Netherlands	Multiple sclerosis	CUA	Early model-based economic evaluation	NA
de Jong et al.^26^	The Netherlands	Inflammatory bowel disease (Crohn’s disease or ulcerative colitis)	CUA	Multicentre RCT (*n* = 4 centres)	909
Gumley et al.^27^	UK, Australia	Service users from community mental health services with recent relapse of psychosis	CUA, CEA	Feasibility cluster RCT (*n* = 8 centres)	73
Seppen et al.^28^	The Netherlands	Rheumatoid arthritis with stable disease activity	CUA, CEA	Single centre non-inferiority RCT	103
Miranda et al.^29^	Canada	People aged 60 years or older with complex chronic conditions (at least two chronic conditions and 10 primary care visits in 12 months)	CUA	Model-based economic evaluation	NA
van der Hout et al.^30^	The Netherlands	Survivors of head and neck cancer, colorectal cancer, breast cancer, or lymphoma	CUA	Multicentre RCT (*n* = 14 centres)	625

*CEA* cost-effectiveness analysis, *CUA* cost-utility analysis, *NA* not applicable, *RCT* randomised controlled trial.

### Logic models summarising the rationale for monitoring

The logic models for the seven active remote monitoring strategies are summarised in Table 2 by their objective, patient inputs, care provider inputs and reported outputs.

**Table 2:** Summary of logic models for each intervention

Author (Year)	Monitoring frequency	Additional patient input	Care provider input	Outputs
Objective: facilitate early intervention (*n* = 4)				
Bernard et al.^24^	Weekly for 6 months	• No additional actions	• Scheduled a 3-month in-person or phone appointment if disease activity was high or data completion was low	• Provider-facing alert if data completion was less than 75% or if responses indicated high disease activity within a standalone dashboard
Cloosterman et al.^25^	As prescribed (typically weekly) for a lifetime	• No additional actions	• Review symptom data between consultations to detect outcome worsening	• Provider-facing graph of longitudinal symptom data within a standalone dashboard
de Jong et al.^26^	3-monthly; Weekly for 12 months	• Increased monitoring frequency if flaring	• Review dashboard twice daily for alerts; • Schedule a follow-up visit, as required, after receiving an alert of high disease activity	• Provider-facing alert if responses indicated high disease activity within a standalone dashboard
Gumley et al.^27^	Daily for 12 months	• No additional actions	• Peer support workers communicated regularly with users to improve engagement; • Escalate care as required after receiving an alert indicating a high-risk of outcome worsening	• Provider-facing graph of longitudinal outcome data within a standalone dashboard; • Provider-facing alert to highlight worsening outcomes within a standalone dashboard and via email; • Patient-facing self-management alerts within the app
Objective: facilitate patient-initiated care (*n* = 1)				
Seppen et al.^28^	Weekly for 12 months	• Contact a nurse upon receiving an alert in the app detecting worsening disease activity if necessary	• View patient-reported data within the electronic medical record if required; • Schedule a nurse appointment when requested to manage worsening disease activity	• Patient-facing graph of longitudinal outcome data within the app; • Patient-facing alert to highlight a flare in disease activity within the app
Objective: facilitate self-management (*n* = 2)				
Miranda et al.^29^	Monthly; Weekly; Daily for 15 months	• Attend a planning appointment to define self-management goals	• Facilitate a planning appointment to set a goal-oriented care plan using a standalone online portal	• Patient-facing graph of longitudinal data via app or standalone dashboard
van der Hout et al.^30^	Patient choice for 6 months	• Decide on the topics to monitor within the app	• No additional actions	• Patient-facing self-management feedback based on responses provided via the app

Four studies used active remote monitoring to alert healthcare staff or patients about symptom worsening and facilitate earlier intervention to improve health outcomes^24–27^. The outcomes that were monitored comprised self-reported disease activity^24,26^, broader quality of life or wellbeing instruments^24,27^ and specific constructs related to the long-term condition, such as fatigue, stress, memory, medication adherence and psychosocial factors^25,26^. The frequency of monitoring ranged between daily to every 3 months. All four examples reported these data using a standalone provider-facing dashboard. In three cases, care providers received an alert if the monitoring data indicated worsening outcomes, which required potential clinical intervention^24,26,27^. In two cases, the longitudinal monitoring data were reported as a provider-facing graph^25,27^.

One study used active remote monitoring to facilitate patient-initiated care by monitoring self-reported disease activity^28^. In this example by Seppen et al., the longitudinal electronic patient-generated health data were assumed to reduce healthcare costs by removing the need for routine outpatient appointments (extending the time to the next in-person follow-up) if the long-term condition remained stable^28^. The monitoring data were integrated into patients’ electronic medical records if providers wished to view responses. A patient-facing graph of their longitudinal data was displayed within the app alongside an alert to indicate a severe increase in disease activity. Patients could contact healthcare providers via the app at any time to schedule a nurse appointment if required. Monitoring was performed weekly for this patient-initiated follow-up intervention.

Two studies used active remote monitoring to facilitate self-management^29,30^. In both cases, patients were first required to decide which outcomes they would like to monitor longitudinally. In the example by Miranda et al., this decision was facilitated by care providers within a planning appointment to define self-management goals^29^. Data were reported as a patient-facing graph of longitudinal responses via the app or a standalone dashboard^29^. In the example by van der Hout et al., by contrast, patients decided on their monitoring outcomes independently^30^. Patient-facing self-management feedback was then displayed within the app based on their responses to the questions^30^. Both examples also asked users to monitor broader constructs affecting quality of life, including physical and psychological wellbeing^29,30^. The frequency of monitoring for these self-management interventions was variable according to patient choice or the self-defined goal of monitoring.

### Cost of mobile device-based active remote monitoring

Two studies used a top-down costing approach to estimate the cost of the intervention^28,30^. Seppen et al. apportioned the yearly cost of their intervention app to the provider centre (€17,500) by the number of patients expected to use the app (20% of patients with rheumatoid arthritis at a single hospital), which resulted in a cost of €17.50 per patient^28^. van der Hout et al. apportioned the cost of running their app (€450,000 per year) by the number of patients expected to use the app (16% of all new cancer cases in the Netherlands), which resulted in a cost of €25 per patient^30^.

Three studies used a bottom-up micro-costing approach to calculate the intervention cost by quantifying the value of each resource item consumed^24,27,29^. As these studies reported resource items at a more granular level, a breakdown of these resources is provided in Table 3 according to the checklist of programme cost components for digital health interventions by Khan et al.^13^. All studies reported cost for the Maintenance, Implementation and Health Personnel Involvement categories; two studies reported costs for the Development category; and no study reported costs for the Research category. Miranda et al. estimated the cost of their intervention by using cost sheets to measure the different activities, quantity of resources consumed and the price of each item prospectively, which resulted in a cost of €1765.93 per patient^29^. The cost of the smartphone-based active remote monitoring intervention in Gumley et al. ranged between £2202 and £2447 per patient, depending on the chosen perspective (healthcare system or societal)^27^. Bernard et al. constructed three cost categories for their intervention: (1) the smartphone app cost which was provided by a vendor and apportioned by the number of participants in the intervention arm; (2) training for participants to use the app; and (3) ongoing support and co-ordination of the smartphone app with a clinical case manager^24^. This resulted in a cost of €914.80 per participant^24^.

**Table 3:** Reported resource items included in the intervention cost

	Study		
Cost category	Bernard et al.^24^	Gumley et al.^27^	Miranda et al.^29^
Development	Not reported	Development of app content; App development and registration as a medical device	App modification and feature development
Research	Not reported	Not reported	Not reported
Maintenance	Maintenance; Data housing; Ongoing support	App hosting; App maintenance	Technical support; Professional services support
Implementation	Hardware, Software; Licencing; User training	Hardware; Staff training; Patient time cost of using app	Technology training; Licencing; Onboarding management
Health Personnel Involvement	Time spent in support and coordination of the app	Routine monitoring of app data; Ongoing staff supervision	Communication with health care teams

Note: Cost categories derived from Khan et al.^13^.

Two studies assumed a fixed cost per user for the cost of their mobile device-based active remote monitoring intervention^25,26^. Cloosterman et al. assumed that the annual cost of their smartphone app was €480 per patient, but no additional details were reported on how this cost was calculated^25^. de Jong et al. assumed that patients who used the smartphone intervention incurred an annual licence fee of €40 per patient^26^.

### Cost-effectiveness results

Table 4 reports the results from the cost-effectiveness analyses for each included study. The base case point estimates indicated that mobile device-based active remote monitoring was cost-effective in six out of seven studies^24–28,30^. These results were dominant (improved health outcomes and reduced costs) in four studies^24,26,27,30^ and improved incremental health outcomes but at acceptable incremental cost in two studies^25,28^. One study indicated that active remote monitoring was not cost-effective for people over 60 years old with complex chronic conditions because it was dominated by multidisciplinary primary care with family health teams (reduced health outcomes and increased costs)^29^.

**Table 4:** Cost-effectiveness results

Study	Primary result	Incremental cost	Incremental health benefit	Currency (Price year)
Trial-based economic evaluations (*n* = 5)				
Bernard et al.^24^	Dominant	Societal perspective: −€72	QALY: 0.07	Euro (2020)
de Jong et al.^26^	Dominant	Societal perspective: −€547 (–€1029 to €2143)	QALY: 0.0020 (−0.022 to 0.018)	Euro (2018)
Gumley et al.^27^	Dominant	Societal perspective: £170 (−£7783 to £8124) Healthcare perspective: −£382 (−£8347 to £7583) Health payer perspective: −£251 (−£8073 to £7571)	QALY: 0.056 (−0.031 to 0.143)	British Sterling (2017)
Seppen et al.^28^	Cost-effective	Societal perspective: €234 (−€3070 to €3438) Healthcare perspective: €12 (–€1232 to €1257)	DAS28: 0.05 (−0.033 to 0.043) QALY: 0.00 (−0.07 to 0.06)	Euro (2020)
van der Hout et al.^30^	Dominant	Societal perspective: −€40 (−€344 to €241) Healthcare perspective: −€163 (−€665 to €326)	QALY: 0.0017 (−0.0121 to 0.0155)	Euro (2017)
Model-based economic evaluations (*n* = 2)				
Cloosterman et al.^25^	Cost-effective	Societal perspective; Effectiveness 5% €6258 Societal perspective; Effectiveness 10%: €3556 Societal perspective; Effectiveness 15%: −€194 Societal perspective; Effectiveness 20%: −€3659 Healthcare perspective; Effectiveness 5% €9183 Healthcare perspective; Effectiveness 10%: €9458 Healthcare perspective; Effectiveness 15%: €8756 Healthcare perspective; Effectiveness 20%: €8358	QALY; Effectiveness 5%: 0.43 QALY; Effectiveness 10%: 0.87 QALY; Effectiveness 15%: 1.33 QALY; Effectiveness 20%: 1.78	Euro (Not reported)
Miranda et al.^29^	Dominated	Healthcare perspective: CAD $1710	QALY: −0.03	Canadian dollar (2020)

*CAD* Canadian dollar, *DAS28* disease activity score 28-joint count, *QALY* quality-adjusted life year.

One study reported their results for a healthcare system perspective only^29^. Two studies reported their results from a societal perspective only^24,26^. Four studies reported their results for both a healthcare system and societal perspective^25,27,28,30^. The non-medical costs included by studies that used a societal perspective comprised labour productivity (*n* = 5 studies)^25–28,30^, travel costs (*n* = 4 studies)^24,26,28,30^, informal care services (*n* = 3)^25,27,30^, time engaging with the mobile device app (*n* = 1)^27^, the criminal justice system (*n* = 1)^27^ and out-of-pocket expenditures (*n* = 1)^27^.

Whilst the base case results from cost-effectiveness analyses are determined by the expected incremental cost and health outcomes, the included studies demonstrated considerable uncertainty with respect to whether the mobile device-based active remote monitoring strategies were cost-effective. A cost-effectiveness plane is used to present the results of an economic evaluation illustrating the joint distribution of incremental costs and incremental health outcomes^31^. For trial-based analyses, this joint distribution is formed by bootstrapping to handle sampling uncertainty^32^. In all five trial-based cost-effectiveness analyses included in this study, the bootstrapped incremental outcomes were distributed across all four quadrants of the cost-effectiveness plane^24,26–28,30^. This phenomenon indicates that the direction of these incremental cost and health outcomes was uncertain (Table 4)^24,26–28,30^. For the model-based early economic evaluation by Cloosterman et al., uncertainty in the relative effectiveness of the intervention strategy was handled by estimating incremental outcomes across a range of plausible treatment effect sizes^25^. For this study, the analysis was deemed to be an early economic evaluation because estimates of intervention’s effectiveness were not yet available.

### Quality assessment

Table 5 reports the assessment of study quality against the CHEERS-2022 reporting criteria for methods and results^33^. The seven included studies are good quality according to the frequency of reported items. The need to specify health economic analysis plans, distributional effects of cost and health outcomes and the effects of engaging with people affected by the study are likely underreported due to the relatively recent introduction of these reporting criteria^33^. No study reported a subgroup analysis to investigate how heterogeneity in population characteristics affected the cost-effectiveness of mobile device-based active remote monitoring.

**Table 5:** Quality assessment of reported methods and results

CHEERS-2022 reporting criteria	Bernard et al.^24^ (2022) ^24^	Cloosterman et al.^25^	de Jong et al.^26^	Gumley et al.^27^	Seppen et al.^28^	Miranda et al.^29^	van der Hout et al.^30^
Methods							
Health economic analysis plan	×	×	×	P	P	×	P
Study population	✓	✓	✓	✓	✓	✓	✓
Setting & location	✓	✓	✓	✓	✓	✓	✓
Comparators	✓	✓	P	✓	✓	✓	✓
Perspective	P	✓	✓	✓	P	✓	✓
Time horizon	✓	✓	✓	✓	✓	✓	✓
Discount rate	P	×	×	✓	✓	P	✓
Selection of outcomes	✓	✓	✓	✓	✓	✓	✓
Measurement of outcomes	✓	✓	✓	✓	✓	✓	✓
Valuation of outcomes	✓	P	✓	✓	✓	✓	✓
Measurement & Valuation of resources & costs	✓	P	✓	✓	✓	✓	✓
Currency, price date, & conversion	✓	×	P	✓	✓	✓	✓
Rationale & description of the model	NA	✓	NA	NA	NA	P	NA
Analytics & assumptions	✓	P	✓	✓	✓	P	✓
Characterizing heterogeneity	×	×	×	×	×	×	×
Characterizing distributional effects	×	×	×	×	×	×	×
Characterizing uncertainty	✓	✓	✓	✓	✓	✓	✓
Approach to engagement with people affected by the study	×	P	×	P	×	P	×
Results							
Study parameters	P	×	✓	✓	✓	✓	✓
Summary of main results	✓	✓	✓	✓	✓	✓	✓
Effect of uncertainty	✓	✓	✓	✓	✓	✓	✓
Effect of engagement with people affected by the study	×	×	×	×	×	×	×

Note: Reporting criteria items from the CHEERS-2022 statement^34^. Notation: ✓: reported fully; P: reported partially; ×: not reported; NA: not applicable.

## Discussion

This study identified seven published cost-effectiveness analyses of mobile device-based active remote monitoring strategies for people with long-term health conditions. The active remote monitoring strategies were deemed to be cost-effective in six of these studies^24–28,30^. In four studies, active remote monitoring was found to be dominant by improving health outcomes (expressed as quality-adjusted life years) and reducing healthcare costs^24,26,27,30^. There was notable uncertainty in the joint distribution of incremental costs and incremental health outcomes, which may preclude these interventions from being recommended widely by decision-makers based on current levels of evidence.

There is growing interest from policymakers internationally to increase the adoption of effective digital health technologies, and the findings from this review provide preliminary support for mobile-based active remote monitoring as an emerging class of digital intervention for people with long-term conditions. Previous reviews have only appraised evidence for the cost-effectiveness of remote monitoring strategies that did not require active user input to track self-reported symptoms or patient-reported outcomes and so identified studies outside the scope of the present review. For example, Guzman et al. reviewed 34 published economic evaluations of non-invasive monitoring devices (excluding those which used self-reported patient data only) and found that these technologies tended to improve health outcomes but also increased costs compared with care as usual^34^. The authors also concluded that cost parameters (such as capital investment) had the greatest influence on whether these monitoring interventions were deemed to be cost-effective^34^. Similarly, Kidholm et al. reviewed nine economic evaluations alongside RCTs of home monitoring equipment for chronic diseases (e.g. spirometer, heart rate or blood pressure monitors) and found that these technologies often led to cost reductions in acute and primary care^35^. Goff-Pronost et al. reviewed sixty-one economic evaluations of telemonitoring interventions (comprising physical devices and remote consultations) and found that 72% were cost-effective compared with care as usual^36^. One key advantage of mobile device-based active remote monitoring strategies, compared with these other examples, is that the high prevalence of device ownership within the general population presents an opportunity to deploy these interventions at scale with a relatively lower upfront cost to the healthcare system^11^. The present study builds on the findings from these earlier reviews to demonstrate that mobile device-based active remote monitoring strategies requiring regular user responses to self-reported questions about their long-term conditions also have the potential to be cost-effective.

The majority of studies in this review used a trial-based design to estimate cost-effectiveness^24,26–28,30^. It is challenging to draw conclusions about why these trial-based designs produced a high degree of uncertainty in their estimates (Table 5). In two trial-based cost-effectiveness analyses, the reported uncertainty was likely due to the trial design (non-inferiority and feasibility trials)^27,28^. In three superiority trial-based designs, the extent of uncertainty may indicate either no difference between the arms or that the trials were underpowered to detect a difference in cost and health outcomes^24,26,30^. This uncertainty may also be reflective of inequalities in digital literacy, if the active remote monitoring interventions were less effective for trial participants who experienced greater barriers to using digital technologies when managing their long-term condition. Alternatively, reduced adherence to monitoring over the trials’ follow-up durations may have reduced its effectiveness compared with care as usual. Decision-analytic models are used routinely to estimate the cost-effectiveness of health technologies, more broadly, but were relatively underutilised within the sample of included studies (*n* = 2 examples)^25,29^. Decision-analytic models can synthesise evidence from different sources and extrapolate outcomes over a longer time horizon (a lifetime horizon is typical for long-term conditions)^37^. There is clear scope to increase the use of decision-analytic models to estimate the cost-effectiveness of future mobile device-based active remote monitoring strategies^16^. These model-based analyses can also be performed at an early stage to support product development by establishing the conditions under which active remote monitoring will be cost-effective^38,39^ (for example, the study by Cloosterman et al.^25^).

The wide variation between the studies in their estimated intervention costs was likely driven by differences in their costing methods (top-down costing; bottom-up micro-costing; assumptions). Estimates informed by top-down costing were substantially lower than by bottom-up micro-costing. This finding may indicate that top-down costing underestimates the costs borne by healthcare systems, if relevant resources or consumables are omitted from the analysis^40^. Cost estimates that rely on apportioning commercial licence fees across patient populations will not necessarily reflect the total cost incurred by a healthcare system to run the service. Similarly, cost estimates that quantify ongoing delivery costs alone may omit important upfront costs such as staff training or infrastructure investments. Emerging frameworks for estimating the cost of digital health interventions provide a useful set of resource items to consider accounting for within cost-effectiveness analyses^13^. Reference to these frameworks alongside micro-costing methods will help to standardise the costs incurred to set-up and deliver digital health technologies within a routine care setting. This standardisation will help healthcare decision-makers compare across studies and improve the usefulness of future cost-effectiveness analyses for mobile device-based active remote monitoring strategies.

The majority of the studies in this review used an external dashboard for presenting the monitoring outputs to healthcare staff. While external dashboards may incur fewer resources to set-up than full integration with electronic healthcare records, they may also impose a burden on healthcare staff when navigating to these dashboards as part of their routine workflow. Future cost-effectiveness analyses of active remote monitoring with greater integration into existing healthcare data systems will need to reflect the set-up cost of this infrastructure investment. Similarly, interventions will need to ensure that they are interoperable with different healthcare data systems to achieve this integration. The expected impact on the healthcare workforce will also differ between clinical applications of mobile-based active remote monitoring strategies. Healthcare decision-makers will need to be aware of the workforce requirements when adopting these digital interventions in the context of existing staffing constraints on healthcare delivery. For app developers and evaluators, establishing a clear logic model to explain how active remote monitoring should lead to health improvements, with an emphasis on the actions required by healthcare staff to facilitate these improvements, will be essential to understand whether it is feasible to integrate these digital technologies within their chosen setting. The actions of healthcare staff can vary between a passive role (such as responding to alerts based on user responses) to a more active role (such as reviewing the monitoring data at regular intervals). This ongoing time commitment required by healthcare providers will also incur a cost per patient that should be quantified in cost-effectiveness analyses of active remote monitoring strategies conducted from a healthcare system perspective.

For decision-makers who require cost evidence from a broader patient perspective beyond those borne by the healthcare system, the included studies highlighted two relevant areas which may be valuable for developers and analysts to consider. The first is the time cost associated with using the smartphone app. Active remote monitoring strategies only work if users engage with regular symptom monitoring, which imposes an opportunity cost to patients on their time to participate in other activities. For example, Gumley et al. valued this time cost at the rate of leisure activity^27^. App developers can design their technologies to minimise this time cost whilst retaining functionality to be useful for remote monitoring. Usage and engagement data can be helpful in this context to understand the time cost to users when accessing the app and completing regular self-reported symptom questions. The second area is the travel costs borne by patients, which was considered by four studies^24,26,28,30^. If active remote monitoring replaces the need for patients to incur out-of-pocket travel costs for scheduled in-person appointments, then these savings accrued to patients could be measured and valued within a cost-effectiveness analysis.

One potential limitation of this study was that Medline and Embase were the only databases searched raising the risk of not finding pertinent CEAs. This risk is likely to be low because a study conducted by information specialists found that the majority of published CEAs can be found in these two electronic databases^41^. A second potential limitation was that there are no established search filters to identify active remote monitoring interventions specifically, so the search terms used by this study may have missed publications that used different words within their titles and abstracts. A third limitation is that while the studies appear to show similar results across different healthcare settings (Australia, Canada, France, The Netherlands, United Kingdom) and intervention designs, the findings may not necessarily generalise to other active remote monitoring interventions or healthcare settings because the outputs from cost-effectiveness analyses are specific to the named healthcare jurisdiction and intervention. When interpreting the evidence from this study, decision-makers should reflect on whether their own healthcare setting or candidate intervention shares similarities with those studies included in this review, and if not, consider undertaking a bespoke analysis for their specific decision problem by building on the methods described by this review.

Future research should consider increasing the use of decision-analytic modelling to extrapolate beyond short-term follow-up durations to estimate the cost and health outcomes delivered by long-term usage of mobile device-based active remote monitoring. This long-term modelling evidence will be especially useful for patient populations who will be expected to engage with remote monitoring over an extended duration or if there is a time-lag between routine monitoring and achieving patient benefit. Future research could also investigate the feasibility of using non-randomised quasi-experimental study designs to estimate clinical and economic endpoints from a smartphone-based active remote monitoring strategy. These study designs would be particularly valuable if the resources required to undertake an RCT are prohibitive for app developers. The cost-effectiveness of different monitoring frequencies for a given clinical application would also be a valuable topic for future research. There did not appear to be a relationship between the frequency of monitoring and its cost-effectiveness across the included studies in this review. However, within a specific clinical setting, differences in monitoring protocols may lead to differences in effectiveness, cost and cost-effectiveness. For decision-makers who wish to explore the impact of mobile-based active remote monitoring strategies beyond a cost-effectiveness analysis from a healthcare system perspective, a social return on investment framework or multi-criteria decision analysis could be used in future research to quantify the costs and benefits accrued to different parties within and beyond the healthcare system. Future research could build on the findings from this review to standardise micro-costing methods for estimating the upfront cost of digital health interventions within routine care settings.

This study found that mobile device-based active remote monitoring strategies have the potential to be cost-effective. However, prevailing levels of decision uncertainty may preclude these strategies from being adopted more widely based on current evidence. Developers and analysts tasked with assessing the value of active remote monitoring strategies can build on the findings of this study by presenting more transparent estimates of upfront cost and a detailed exploration of the parameters contributing to decision uncertainty to help to strengthen the case for adopting these digital health technologies. In doing so, people with long-term conditions will ultimately benefit from access to effective and cost-effective mobile device-based active remote monitoring strategies that will improve their management over time.

## Methods

A systematic literature review was performed to identify all published cost-effectiveness analyses of a mobile device-based active remote monitoring strategy for people with long-term conditions. The protocol was registered via the International Prospective Register of Systematic Reviews (PROSPERO) [ID Number: CRD42023406364]. The study is reported according to the Preferred Reporting Items for Systematic Reviews and Meta-analyses (PRISMA) 2020 statement^23^. A completed PRISMA statement is available in Supplementary File 1.

### Eligibility criteria

The criteria for inclusion to this study are reported in Table 6. The relevant patient population comprised individuals with at least one long-term condition (diagnosed clinically or self-reported). The relevant intervention comprised any mobile device-based active remote monitoring strategy that used a mobile device (smartphone or tablet). These interventions required patients to self-report answers to a repeated series of questions about their long-term condition over time (i.e. respond to the same questions more than once over any time duration). Questions were required to measure any dimension of health, including patient-reported outcome measures (such as self-reported symptoms or quality of life) and clinical outcomes (such as self-completed disease activity scores). No restrictions were placed on the clinical rationale for monitoring, the degree of integration with healthcare provider infrastructure, or whether the intervention was delivered by an app on the mobile device or web browser. This definition excluded interventions that required monitoring outputs or measurements from an additional physical device (for example, blood pressure cuff or spirometer), or passive remote monitoring technologies that used wearable sensors instead of active user self-reported measurements (for example, physical activity measured through a wrist-worn fitness tracker). The relevant study design was a cost-effectiveness analysis, defined as a method involving an incremental comparison of alternative courses of action in terms of their cost and health consequences^20^. Cost-effectiveness analyses could express their health consequences in natural units or preference-based health utility values (also known as cost-utility analyses).

**Table 6:** Study inclusion criteria

Element	Description
Population	• Adults with at least one long-term condition (diagnosed clinically or self-reported)
Intervention	• Any mobile device-based active remote monitoring strategy which used a smartphone or tablet;
	• Users were required to self-report responses to a repeated series of questions;
	• Questions could refer to any aspect of their long-term condition (for example: symptoms, clinical outcomes, patient-reported outcomes, quality of life outcomes);
	• Interventions were excluded if they measured responses via a standalone physical device only or if they comprised a passive remote monitoring device only.
Comparator	• Any relevant comparator strategy
Outcome	• Incremental cost, incremental health outcomes, incremental cost-effectiveness
Study design	• Full economic evaluation (cost-effectiveness analysis or cost-utility analysis) using a trial-based or decision-analytic model-based design in a peer-reviewed journal (language: English)

### Information sources and search strategy

Medline (and Epub Ahead of Print, In-Process, In-Data-Review and Other Non-Indexed Citations and Daily) and Embase were searched electronically via Ovid from 2008 until 8 November 2024. These search dates were chosen to coincide with the launch of the online application marketplaces for mobile devices in 2008^42^. Medline and Embase were appropriate databases to search because earlier studies found that the majority of published economic evaluations were indexed on these platforms^41^. The electronic database search was supplemented by hand-searching the reference lists of included studies. The search strategy comprised terms for digital health technologies, active remote monitoring of user-reported outcomes and cost-effectiveness analyses (reported in Supplementary File 2). These search terms were adapted from the terms used by other published systematic reviews of digital health technologies^34,43^.

### Selection process

The titles and abstracts were screened against the eligibility criteria by two authors independently (SPG, NB). Cohen’s kappa was calculated to indicate the degree of agreement between the reviewers^44^. Disagreements at this stage were resolved by including studies for full text review if at least one author judged that the title and abstract met the eligibility criteria. Automation tools were not used for study selection. Candidate studies were read in full by two authors (SPG, NB) to determine if they met the eligibility criteria. Disagreements were resolved by discussion.

### Data extraction and analysis

The following data items were extracted from each included study by one author (SPG): author, year of publication, country of analysis, target population of patients, description of the mobile device-based active remote monitoring strategy, description of the comparator strategy, type of economic evaluation (cost-effectiveness analysis or cost-utility analysis), study design (trial or decision-analytic model-based analysis), primary clinical endpoint, estimated incremental cost, estimated incremental health outcomes, estimates of cost-effectiveness (if reported) and key uncertainties driving estimates of cost-effectiveness.

Data were reported in a table and summarised by a narrative synthesis. Meta-analysis was not performed because it is not appropriate to quantitatively synthesise results from cost-effectiveness analyses^45^. The overall study designs were summarised first with an emphasis on understanding the long-term conditions served by the active remote monitoring strategy and whether these target populations were experiencing ongoing symptoms or stable activity.

To help distinguish how each active remote monitoring strategy was hypothesised to deliver benefit, the logic model for each intervention strategy was summarised by its component parts using the following subheadings: Objective (to define the overall goal of active remote monitoring); Inputs (to define the necessary patient and care provider actions to operationalise active remote monitoring); and Outputs (to define how the patient-reported data were presented or used to achieve the desired objective)^46,47^. If the logic model was not available within the included study, then reference was made to the study’s protocol or primary outcome publications (if reported separately from the cost-effectiveness analysis) for this information.

A detailed analysis of the estimated cost associated with each mobile device-based active remote monitoring strategy was then undertaken to explore the variation and drivers of the upfront cost to care providers. The approaches to estimate these intervention costs were first categorised by the methods employed: top-down (gross costing); bottom-up (micro-costing); or assumption-based estimates^48,49^. Studies that estimated the cost of their intervention using bottom-up micro-costing were evaluated further by charting the resource items against the checklist of programme cost components for digital health interventions by Khan et al.^13^. (Cost Categories: Development; Research; Maintenance; Implementation; and Health Personnel Involvement). This charting activity was possible because bottom-up micro-costing methods provide a clear breakdown of resources contributing to cost estimates^49^.

Finally, the estimated cost-effectiveness of the mobile device-based active remote monitoring strategies was assessed. A table summarised the estimated incremental cost (with currency and the reported price year) and incremental health outcomes for each intervention alongside the corresponding estimates of uncertainty (if reported). The base case (primary outcome) estimate of cost-effectiveness was categorised as Dominant (if the intervention improved health outcomes and reduced cost), Dominated (if the intervention worsened health outcomes and increased cost), or Cost-effective (if the ratio of incremental costs to incremental health outcomes was below the reported threshold to define cost-effectiveness)^20^. For completeness, the results for different scenario analyses and different costing perspectives (healthcare system or societal perspective) were also reported if included by each study.

### Quality assessment

The 2022 Consolidated Health Economic Evaluation Reporting Standards (CHEERS-2022) statement was used to appraise the quality of reporting the methods and results for each study included in the review^33^. Publication bias was not assessed. Two authors (SPG; ACTT) determined if the 22 items associated with the methods and results were reported fully, partially, or not at all. The results of the quality assessment were reported in a table to establish patterns of reporting across the sample of included studies.

## Supplementary information

Supplementary information

## Data Availability

All data included in this systematic review are available publicly via the seven original published studies which can be accessed online. Direct links to these seven published studies can be found in the reference list (references 24–30). Additional datasets were not generated or analysed for this study.

## Code availability

The search strategy for this systematic review is reported open access in Supplementary File 2. The search was performed online via the Ovid platform (date: 8 November 2024).
